# Supportive care needs among head and neck cancer patients in the recovery phase from 6 months to 2 years after treatment: which factors matter?

**DOI:** 10.1007/s11764-025-01753-0

**Published:** 2025-02-10

**Authors:** D. Molenaar, I. M. Verdonck-de Leeuw, B. I. Lissenberg-Witte, R. P. Takes, R. de Bree, J. A. Langendijk, J. A. Hardillo, F. Lamers, C. R. Leemans, F. Jansen

**Affiliations:** 1 Cancer Center Amsterdam Research Institute, Amsterdam UMC, Vrije Universiteit Amsterdam, Amsterdam, The Netherlands; 2 Department of Otolaryngology-Head and Neck Surgery, VUmc Cancer Center Amsterdam, Amsterdam UMC Location VUmc, De Boelelaan 1117, 1081 HV Amsterdam, The Netherlands; 3 Department of Clinical, Neuro- and Developmental Psychology, Faculty of Behavioral and Movement Sciences & Amsterdam Public Health Research Institute, Vrije Universiteit Amsterdam, Van Der Boechorststraat 7, 1081 BT Amsterdam, The Netherlands; 4 Department of Epidemiology and Data Science, Vrije Universiteit Amsterdam, Amsterdam UMC, Amsterdam, The Netherlands; 5 Department of Otorhinolaryngology and Head and Neck Surgery, Radboud University Medical Center, Nijmegen, The Netherlands; 6 Department of Head and Neck Surgical Oncology, University Medical Center Utrecht, Utrecht, The Netherlands; 7 Department of Radiation Oncology, University of Groningen, University Medical Center Groningen, Groningen, The Netherlands; 8 Department of Otolaryngology and Head and Neck Surgery, Erasmus MC Cancer Institute, Rotterdam, The Netherlands; 9 Department of Psychiatry, Amsterdam Public Health Research Institute, Amsterdam UMC, Amsterdam, The Netherlands; 10 Amsterdam Public Health, Mental Health Program, Amsterdam, The Netherlands

**Keywords:** Head and neck cancer, Supportive care needs, Survivorship care needs, Long-term outcomes, Longitudinal cohort

## Abstract

**Purpose:**

To investigate which demographic, personal, clinical, physical, psychological, social, lifestyle, and cancer-related quality of life (QoL) factors are associated with (changes in) supportive care needs (SCNs) from 6 months to 2 years after treatment in head and neck cancer (HNC) patients.

**Methods:**

Data from the prospective NETherlands QUality of life and BIomedical Cohort (NET-QUBIC) study among HNC patients treated with curative intent was used. SCNs were measured using the Supportive Care Needs Survey 34-item Short-Form (SCNS-SF34) (6 months, 1 and 2 years after treatment) and the 11-item HNC-specific module (SCNS-HNC) (2 years). Multivariable linear mixed model analyses and linear regression analyses were used to study factors associated with changes in SCNs over time (SCNS-SF34) and the level of SCNs at 2 years follow-up (SCNS-SF34 and SCNS-HNC).

**Results:**

Data from 483 patients was used. SCNs in the physical and daily living (PDL), psychological (PSY), and health system, information, and patient support (HSIPS) domains decreased significantly over time. At 2 years follow-up, the highest SCNs were reported regarding lack of energy/tiredness (10.8%). Changes in SCNs and the absolute level of SCNs at 2 years were associated with personal and clinical factors and post-treatment (6 months) with psychological, lifestyle, and cancer-related QoL factors.

**Conclusions:**

Personal, clinical, psychological, lifestyle, and cancer-related QoL factors were associated with SCNs. These results can be used to develop predictive models to personalize supportive care for HNC patients.

**Implications for Cancer Survivors:**

SCNs decrease over time, but a subgroup of patients still presents with SCNs 2 years after treatment.

**Supplementary Information:**

The online version contains supplementary material available at 10.1007/s11764-025-01753-0.

## Introduction

Supportive care needs (SCNs), i.e., the needs for additional care for the prevention and management of adverse effects of cancer and its treatment [1], are widely present among head and neck cancer (HNC) patients [2, 3]. At the time of diagnosis, HNC patients often have a need for information (e.g., being informed about things you can do to help yourself get well) and psychological support (e.g., uncertainty about the future) [4–9], whereas after treatment, they often have a need for support on HNC- and treatment-related symptoms (e.g., problems with a dry mouth) and physical and daily living issues (e.g., pain management). Also, although less often reported, HNC patients may express needs for support in the field of sexuality and lifestyle [8, 10–15]. To improve tailoring of supportive care to the individual patient, detailed insight is needed into groups of HNC patients that show high SCNs (i.e., absolute scores) as well as those who show limited improvement or worsening of SCNs over time (i.e., relative scores).

In the last decades, several studies have been performed regarding the absolute level of SCNs [3, 6, 10, 11, 15–20]. These studies focused on HNC patients with mixed tumor locations (e.g., oral cavity, pharynx, and larynx) [3, 11, 15, 18–20] or oral cancer patients only [6, 10, 17]. Studies mostly focused on newly-diagnosed HNC patients [3, 6, 15, 17, 20], whereas three studies included both HNC patients newly diagnosed and those with cancer recurrence [11, 18, 19]. Findings of these studies showed that shorter time since treatment [3, 10, 11, 17, 18], more extensive tumor treatment [6, 11], and a higher level of psychological and HRQOL symptoms after treatment [3, 6, 10, 17–20] are associated with higher SCNs. Conflicting results have been found on age, gender, and tumor stage [3, 4, 6–8, 10, 11, 15–18, 20, 21]. All these studies, however, had a cross-sectional design [3, 6, 10, 11, 15–20], and some also focused on HNC patients with a mixed time interval since diagnosis or treatment [10, 11, 15, 18, 19], limiting the ability to identify groups of HNC patients at risk for SCNs at a specific later time point.

With regard to changes in SCNs over time, Chen et al. [7] previously investigated changes in SCNs from cancer diagnosis up to 6 months follow-up among 82 oral cavity cancer patients. In addition, we recently conducted a longitudinal study among a large cohort of 563 HNC patients from the time of diagnosis (before the start of treatment) up to 2 years after treatment [8]. The aim was to investigate which factors at the time of diagnosis (how patients enter the cancer trajectory) are associated with changes in SCNs over time from the time of diagnosis to 2 years after treatment. On average, SCNs diminished over time. Nevertheless, 43% of HNC patients reported unmet SCNs 2 years after treatment. Changes in SCNs within the first 2 years after treatment were significantly associated with demographic (gender), personal (seeking social support), clinical (tumor site, stage, and treatment), physical (muscle strength), psychological (fear of cancer recurrence), social (social support), lifestyle (smoking and alcohol consumption), and HNC-specific quality of life (QOL) factors, as assessed at the time of HNC diagnosis. Further insight is needed on the association between factors assessed 6 months after treatment (how they overcome the treatment phase) and changes in SCNs from 6 months to 2 years after treatment. Baseline physical, psychological, social, and cancer-related quality of life symptoms can resolve after cancer treatment, become persistent, or increase due to HNC treatment or tumor progression [22–25]. Post-treatment physical, psychological, social, and HNC-specific symptoms may, consequently, explain changes in SCNs in the recovery phase after treatment better than baseline scores.

The aim of this paper is twofold: a) to investigate which demographic, personal, clinical, and post-treatment (6 months after treatment), physical, psychological, social, lifestyle, and cancer-related quality of life factors are associated with changes in SCNs from 6 months to 2 years after treatment and b) to investigate which demographic, personal, clinical, and post-treatment physical, psychological, social, lifestyle, and cancer-related quality of life factors are associated with the level of SCNs at 2 years after treatment.

## Materials and methods

### Design and study population

Patients newly diagnosed with HNC in seven private not-for-profit hospitals in the Netherlands were asked to participate in the NETherlands QUality of life and BIomedical Cohort (NET-QUBIC) study, a longitudinal cohort study with an enrollment of patients from April 2014 until July 2018 [26, 27]. Inclusion criteria were age > 18 years, tumor in the oral cavity, oropharynx, hypopharynx, larynx or unknown primary site, treatment with curative intent, and the ability to write and read Dutch. Exclusion criteria were other tumors in the head and neck area, recurrent or second primary HNC tumors, inability to understand the questions or test instructions, severe psychiatric comorbidities, and inability to understand informed consent [26]. In total, 739 HNC patients were included before the start of treatment. Patients were asked to fill in patient-reported outcome measures (PROMs) before the start of treatment (baseline, T0), at 3 (M3) and 6 months (M6), and at 1 year (M12) and 2 years after treatment (M24). Home visits consisting of an interview, physical tests, and biobank sampling were conducted at T0, M6, M12, and M24. Clinical data was collected through digital patient files. For this study, we used NET-QUBIC data collected at M6, M12, and M24, supplemented by baseline (seen as static) factors on demographic, personal, and clinical factors. All patients included in the NET-QUBIC cohort signed informed consent. The local ethical committee approved the study (Amsterdam UMC, location VUmc, document number: 2013.301[A2018.307]-NL45051.029.13). Only outcome measures relevant to the current study are described. For detailed information on NET-QUBIC, see previous publications [26, 28]. Patients were included in the current study if they had data available on at least one of the SCNS domains at M6.

### Supportive Care Needs Survey (SCNS-SF34)

The Supportive Care Needs Survey (SCNS-SF34) and the HNC-specific module (SCNS-HNC) were used to measure SCNs among HNC patients [29–31]. The SCNS-SF34 consists of 34 items on physical and daily living needs (PDL), psychological needs (PSY), sexuality needs (SEX), and health system, information, and patient support needs (HSIPS) [31]. SCNS-SF34 data was measured at 6-, 12-, and 24-month follow-up. The SCNS-HNC contains 11 items on HNC-specific functioning needs (HNC-function) and lifestyle needs (HNC-lifestyle), and one single item on care of stoma and/or voice prosthesis (results on this single item are not presented in this study). SCNS-HNC data was measured at 24-month follow-up. All items are answered on a 5-point scale: “1 = not applicable” for issues that are no problem to the patient, “2 = satisfied” for issues on which a patient needs support but the support is already satisfactorily fulfilled, and 3, 4, and 5 for issues on which patients reported a low, moderate, or high need for additional care. All questions are answered with regard to the last month and converted to a score ranging from 0 (no SCNs) to 100 (high SCNs on all items). Less than 7% of data on the SCNS-SF34 and SCNS-HNC was missing on the item level. Missing data on the SCNS-SF34 and SCNS-HNC was imputed by the mean score of the other items of the particular domain in case < 50% of all items within the domain were missing. In addition, per item, all patients were dichotomized into having moderate or high unmet (score 4 or 5) SCNs (yes/no).

### Factors studied in relation to supportive care needs

Detailed information on all factors is provided in the NET-QUBIC data catalog [32]. Factors studied at T0 were demographic, personal, and clinical factors. Demographic factors consisted of age, gender, living situation (living alone or living together), educational level according to the standard classification of education level in the Netherlands [33] (lower or primary, secondary or higher education), and employment status (unemployed or employed). Personal factors consisted of personality (NEO Five Factor Inventory, a higher score indicates a higher level of neuroticism, extraversion, agreeableness, conscientiousness, or openness to experience) [34] and coping (Utrecht Coping List, a higher score indicates more active coping, palliative coping, avoidance coping, seeking support, passive coping, expression of emotions, and comforting thoughts) [35]. Clinical factors consisted of treatment (single or multimodality treatment), ICD-10 tumor stage using TNM 7 classification (I, II, III, or IV), tumor site (oral cavity, oropharynx human papillomavirus (HPV) positive, oropharynx HPV negative, oropharynx HPV unknown, hypopharynx, larynx, and unknown primary), comorbidity (none, mild, moderate, or severe using the Adult Comorbidity Evaluation 27 (ACE-27) [36]), and WHO performance score (WHO 0 or WHO I/II/III).

Factors studied at M6 were personal, physical, psychological, social, and lifestyle factors and cancer-related quality of life symptoms. Personal factors consisted of self-efficacy (Generalized Self-efficacy Scale, a higher score indicates better self-efficacy). Physical factors consisted of muscle strength, measured using a JAMAR handgrip dynamometer for the upper extremities [37] and dichotomized into < 10th percentile and ≥ 10th percentile based on values set by the Nutritional Assessment Platform taking the highest grip strength value [38]. Psychological factors consisted of symptoms of anxiety and depression (Hospital Anxiety and Depression Scale (HADS), a higher score indicates higher levels of distress, anxiety, or depression) [39] and fear of cancer recurrence (Cancer Worry Scale (CWS), a higher score indicates higher levels of fear of cancer recurrence) [40]. Social factors consisted of social support (Social Support List–Interactions, a higher score indicates better social support) [41]. Lifestyle factors consisted of smoking and alcohol usage and body mass index (BMI). Smoking and alcohol usage were measured using study-specific questionnaires. Patients were categorized into the smoking group in case they currently smoked every day. Alcohol usage was scored as excessive when consumption exceeded 14 glasses per week for women and 21 for men [42]. Cancer-related quality of life symptoms were measured using the European Organization for Research and Treatment of Cancer (EORTC) core and HNC-specific quality of life module (EORTC QLQ-C30 and EORTC QLQ-H&N35). The EORTC QLQ-C30 consists of a global quality of life scale: five functional scales: physical, role, emotional, cognitive, and social functioning; three symptom scales: nausea and vomiting, fatigue, and pain; and six single items: dyspnea, insomnia, loss of appetite, constipation, diarrhea, and financial problems [43, 44]. The EORTC QLQ-H&N35 comprises seven symptom scales: oral pain, swallowing, senses, speech, social eating, social contact, and sexuality and eleven symptom items: problems with teeth, dry mouth, opening the mouth, sticky saliva, cough, feeling ill, weight loss, weight gain, use of nutritional supplements, feeding tubes, and painkillers [45]. All EORTC scores are converted to a score ranging from 0 to 100. Higher scores on the functioning domains indicate better functioning, and higher scores on the symptom domains indicate higher symptom burden.

### Statistical analysis

Statistical analysis was performed using IBM SPSS, version 28. Outcomes were described using frequencies and percentages or mean and standard deviation (SD). To investigate possible differences between included and excluded patients, independent sample *t*-tests and chi-square tests were performed. To evaluate changes in SCNS-SF34 domains from 6 months to 2 years after treatment, linear mixed models were estimated, with fixed effects for time (categorical variable), and random intercepts at the patient level. A Bonferroni correction was applied to correct for multiple testing; changes over time were significant when *p* < 0.01. To investigate factors associated with changes in SCNs from 6- to 24-month follow-up, a multivariable linear mixed model was built (compound symmetry matrix) per SCNS-SF34 domain, adding the potential factor and time*factor to the model. Time*factor variables were added to the multivariable mixed model using forward selection. A *p*-value < 0.05 was used in these multivariable linear mixed models, as the aim of this study was to conduct exploratory analyses [46]. Changes were only investigated for the SCNS-SF34 domains, as the SCNS-HNC was not completed at 6 and 12 months. To investigate factors associated with the absolute level of SCNs (both SCNS-SF34 and SCNS-HNC) at 2 years follow-up a linear regression model was estimated per domain using forward selection (*p* < 0.05).

## Results

Of the 739 patients who participated in the NET-QUBIC cohort, 31 died before M6. Of the 708 patients alive at M6, 483 patients completed at least one domain of the SCNS-SF34 or SCNS-HNC and were included in the study. The mean age of the study population was 63.8 (SD 9.4) years, and the majority (74.5%) was male. Most patients presented with an oropharynx tumor (*N* = 171, 35%), followed by a larynx tumor (*n* = 137, 27.7%) and an oral cavity tumor (*N* = 131, 27.1%). Patients most often had advanced staged tumors; 196 patients (40.6%) presented with a stage IV tumor. Over half of the patients received single treatment (*n* = 265; 54.9%) consisting of surgery or radiotherapy. Patients included in this study (*n* = 483) differed from the excluded patients (*n* = 225) regarding educational level (lower educated patients in the excluded group), comorbidities (more patients with higher comorbidity scores in the excluded group), and WHO status (more patients with higher WHO status in the excluded group) (Table 1).

**Table 1:** Characteristics of included patients compared to excluded patients

Characteristics	Included patients (*N* = 483)	Excluded patients (*N* = 225)	*p*-value
Age (SD)	63.8 (9.4)	61.7 (10.3)	*p* = 0.091
Gender			*p* = 0.830
Male (%)	360 (74.5)	166 (73.8)	
Female (%)	123 (25.5)	59 (26.2)	
Educational level^a^			*p* = 0.003
Lower or primary (%)	176 (38.4)	86 (52.1)	
Secondary (%)	125 (27.3)	43 (26.1)	
Higher (%)	157 (34.3)	36 (21.8)	
Missing	25	60	
Tumor site			*p* = 0.197
Oral cavity (%)	131 (27.1)	57 (25.3)	
Oropharynx HPV positive (%)	95 (19.7)	34 (15.1)	
Oropharynx HPV negative (%)	52 (10.8)	37 (16.4)	
Oropharynx HPV unknown (%)	24 (5.0)	9 (4.0)	
Hypopharynx (%)	30 (6.2)	18 (8.0)	
Larynx (%)	137 (27.7)	66 (29.3)	
Unknown primary (%)	17 (3.5)	4 (1.8)	
Tumor stage			*p* = 0.155
I (%)	121 (25.1)	41 (18.2)	
II (%)	89 (18.4)	39 (17.3)	
III (%)	77 (15.9)	45 (20.0)	
IV (%)	196 (40.6)	100 (44.4)	
Treatment^b^			*p* = 0.411
Single (%)^c^	265 (54.9)	116 (51.6)	
Multiple (%)^d^	218 (45.1)	109 (48.4)	
Comorbidities			*p* < 0.001
None (%)	151 (32.5)	49 (21.8)	
Mild (%)	186 (40.1)	70 (31.1)	
Moderate (%)	83 (17.9)	65 (28.9)	
Severe (%)	44 (9.5)	23 (10.2)	
Missing	19	18	
WHO status^e^			*p* = 0.019
Having no restrictions (%)	353 (73.1)	145 (64.4)	
Having any type of restrictions (%)	130 (26.9)	80 (35.6)	

^a^Lower or primary educational level (± ≤ 13 years of education) includes primary education, lower or preparatory vocational education, and intermediary general secondary education. Secondary educational level (between ± 13 and 17 years of education) includes senior general secondary education and higher general secondary education. Higher educational level (± ≥ 17 years of education) includes higher professional education and university

^b^One patient died before surgery

^c^Single treatment = surgery or CO_2_ laser or radiotherapy

^d^Multiple treatment = chemoradiotherapy or surgery and radiotherapy or surgery and chemoradiotherapy or radiotherapy and hyperthermia

^e^Having no restriction = WHO 0, having any type of restrictions = WHO I, II, and III. No patient had WHO IV score

Between M6 and M24, patients dropped out due to mortality (*n* = 50), physical (*n* = 12), psychological (*n* = 9), or logistic reasons (*n* = 8), no more willingness to participate (*n* = 3), and continuation of treatment in a non-participating center (*n* = 1) (Appendix 1).

### Supportive care needs from 6 months to 2 years after treatment

**Fig. 1:**
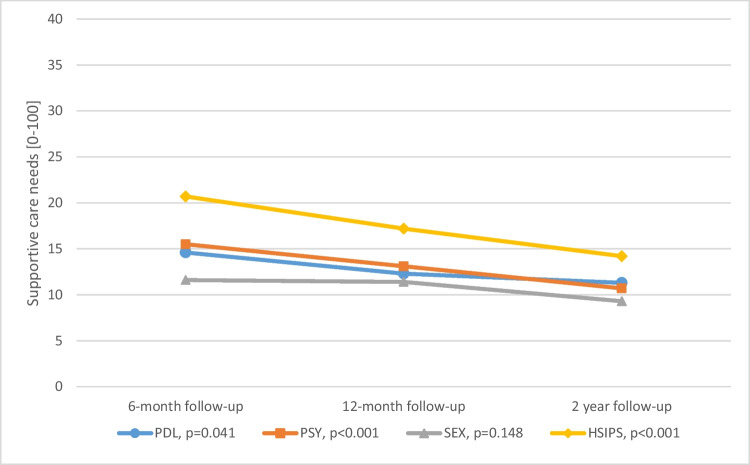
Changes in supportive care needs from 6 months to 2 years follow-up

The SCNs decreased significantly over time for the SCNS-SF34 domains: PDL, PSY, and HSIPS. SEX needs did not change over time (Fig. 1, Appendix 2). At M24, the top 5 of both SCNS-SF34 and SCNS-HNC items were “lack of energy/tiredness (PDL)” (10.8%), “having one member of hospital staff with whom you can talk to about all aspects of your condition, treatment and follow-up (HSIPS)” (9.7%), “being treated like a person not just another case (HSIPS)” (8.5%), “being informed about things you can do to help yourself get well (HSIPS)” (8.4%), and “problems with dry mouth and/or sticky saliva (HNC-specific)” (8.4%) (Appendix 3). Of all patients with complete data at 2-year follow-up (*n* = 157), 40.8% had 1 or more unmet needs. Of these patients (*n* = 64), 25.0% had 1 unmet need, 12.5% had 2 unmet needs, and 62.5% had ≥ 3 unmet needs.

### Post-treatment factors associated with changes in supportive care needs from 6 months to 2 years after treatment

Post-treatment factors (assessed at 6 months) associated with changes in SCNs are presented in Table 2 and Appendix 4. Changes in SCNs among subgroups of HNC patients were, based on visual inspection (Appendix 5), defined as “worse,” when they showed a smaller reduction in SCNs or a larger increase in SCNs over time, in comparison to the reference/comparison group.

**Table 2:** Multivariable linear mixed model results on the *p*-value of the M6 factor*time per SCNS domain

	Physical and daily living	Psychological	Sexuality	Health system, information, and patient support
Personal				
UCL Neuroticism			*p* = 0.042	
UCL Extraversion		*p* = 0.035		
Active tackling	*p* = 0.074			
Clinical				
Type of treatment			*p* = 0.034	
Tumor stage		*p* = 0.004		*p* = 0.017
WHO performance			*p* = 0.015	
Psychological				
Anxiety		*p* = 0.013		
Fear of recurrence		*p* < 0.001		*p* = 0.052
Lifestyle				
Muscle strength			*p* = 0.021	
Smoking			*p* = 0.038	
Symptom specific				
Global quality of life				*p* = 0.011
Physical functioning		*p* = 0.001		
Role functioning		*p* = 0.020		
Cognitive functioning			*p* < 0.001	
Social functioning	*p* = 0.039			
Insomnia	*p* = 0.012			*p* = 0.005
Constipation		*p* < 0.001		
Diarrhea			*p* = 0.002	
Oral pain	*p* = 0.041			
Trouble with social eating				*p* = 0.090
Trouble with social contact	*p* = 0.007			*p* = 0.022
Less sexuality	*p* = 0.004			
Opening mouth		*p* = 0.041		
Sticky saliva				*p* = 0.047
Feeding tube usage	*p* = 0.010	*p* < 0.001		
Weight gain	*p* < 0.001	*p* = 0.005		

Changes in PDL were significantly associated with social functioning, insomnia, oral pain, trouble with social contact, less sexuality, feeding tube usage, and weight gain, as measured at 6 months after treatment. The graphs presented in Appendix 5 show that changes in PDL needs were worse for patients with better social functioning, more insomnia, less oral pain, less trouble with social contact, more sexuality problems, those who used a feeding tube, and those who had no weight gain.

Changes in PSY needs were significantly associated with extraversion, tumor stage, anxiety, fear of cancer recurrence, physical functioning, role functioning, constipation, mouth opening, feeding tube usage, and weight gain. Changes in PSY needs were worse for patients with lower levels of extraversion, high tumor stage, more anxiety, less fear of cancer recurrence, lower levels of physical functioning, better levels of role functioning, less constipation, more trouble with mouth opening, patients with a feeding tube, and those who had no weight gain.

Changes in SEX needs were significantly associated with neuroticism, type of treatment, WHO performance score, grip strength, daily smoking, cognitive functioning, and diarrhea. Changes in SEX needs were worse for patients with lower levels of neuroticism, those treated using multiple treatment, a WHO score of 1 or higher (i.e., any type of restrictions), low grip strength, non-smokers, and those with higher levels of cognitive functioning and more diarrhea.

Changes in HSIPS needs were significantly associated with tumor stage, fear of cancer recurrence, global quality of life, insomnia, trouble with social contact, and sticky saliva. Changes in HSIPS needs were worse for patients with more advanced tumor stages, less fear of cancer recurrence, a lower level of global quality of life, more insomnia, less trouble with social contact, and less sticky saliva.

### Post-treatment factors associated with supportive care needs at 2 years after treatment

Table 3 provides the multivariable outcomes on post-treatment factors (assessed at 6 months) associated with higher levels of SCNs at 2 years after treatment. Post-treatment factors associated with higher levels of PDL needs were higher levels of anxiety, lower levels of role functioning, and higher levels of fatigue, pain, and feeding tube usage. Post-treatment factors associated with higher levels of PSY needs were neuroticism, tumor stage III, a higher level of anxiety and fear of cancer recurrence, a lower level of social functioning, feeding tube usage, and those with no weight gain. Post-treatment factors associated with higher SEX needs were male gender, higher levels of anxiety, lower levels of social functioning, and painkiller usage. Post-treatment factors associated with HSIPS needs were neuroticism, extraversion, a higher level of fear of cancer recurrence, and a lower level of role functioning and social functioning. Post-treatment factors associated with higher HNC-specific functioning needs were less openness, a higher level of seeking social support, a higher level of appetite loss, less constipation, and a higher level of swallowing problems, sexuality problems, and painkiller usage. Finally, post-treatment factors associated with higher HNC-lifestyle needs were tumor location (patients with HPV-positive oropharyngeal tumors had more needs), being a daily smoker, a lower level of emotional functioning, a higher level of trouble with social contact and problems with teeth, a lower level on dry mouth, and a higher level of coughing.

**Table 3:** Factors associated with supportive care needs 2 years after treatment

	Physical and daily living β (95%CI)	Psychological β (95%CI)	Sexuality β (95%CI)	Health system, information, and patient support β (95%CI)	HNC-specific functioning β (95%CI)	HNC lifestyle β (95%CI)
Demographic						
Gender,						
Male			Ref			
Female			− 7.852 (− 12.706 to − 2.999)*			
Educational level,						
Lower or primary						Ref
Secondary						1.662 (− 0.809 to 4.133)
Higher						4.787 (− 0.105 to 9.679)
Personal						
Neuroticism		0.398 (0.158 to 0.638)*		0.633 (0.302 to 1.193)**		
Extraversion				0.461 (0.112 to 0.810)*		
Openness					− 0.482 (− 0.862 to − 0.101)*	
Seeking social support					1.202 (0.541 to 1.864)**	
Clinical						
Tumor site,						
Oral cavity						Ref
Oropharynx HPV +						0.501 (− 0.453 to 1.455)
Oropharynx HPV −						1.644 (0.227 to 3.060)*
Hypopharynx						0.864 (− 1.510 to 3.238)
Larynx						0.074 (− 1.768 to 1.917)
Unknown primary						1.896 (− 3.295 to 7.087)
Oropharynx HPV unknown						− 3.451 (− 13.060 to 6.158)
Tumor stage,						
Stage I		Ref				
Stage II		0.108 (− 1.423 to 1.638)				
Stage III		3.067 (0.676 to 5.458)*				
Stage IV		3.241 (− 0.811 to 7.294)				
Psychological						
Anxiety	1.127 (0.565 to 1.690)**	1.337 (0.673 to 2.001)**	1.225 (0.492 to 1.957)*			
Fear of recurrence		0.792 (0.245 to 1.339)*		0.613 (0.034 to 1.193)*		
Lifestyle						
Smoking						12.246 (5.652 to 18.839)**
Symptom specific						
Role functioning	− 0.139 (− 0.245 to − 0.033)*			− 0.130 (− 0.243 to − 0.017)*		
Emotional functioning						− 0.137 (− 0.268 to − 0.006)*
Social functioning		− 0.185 (− 0.274 to − 0.095)**	− 0.127 (− 0.244 to − 0.010)*	− 0.159 (− 0.284 to − 0.035)*		
Fatigue	0.153 (0.052 to 0.254)*					
Appetite loss					0.139 (0.039 to 0.239)*	
Constipation					− 0.151 (− 0.265 to − 0.036) *	
Pain	0.139 (0.035 to 0.243)*					
Swallowing		0.084 (− 0.017 to 0.184)			0.216 (0.068 to 0.363)*	
Trouble with social eating					0.147 (− 0.021 to 0.315)	
Trouble with social contact						0.290 (0.051 to 0.528)*
Less sexuality					0.114 (0.051 to 0.178)**	0.055 (− 0.010 to 0.119)
Teeth						0.149 (0.054 to 0.243)*
Dry mouth						− 0.110 (− 0.177 to − 0.044)*
Coughing						0.102 (0.014 to 0.189)*
Painkillers, No			Ref		Ref	
Yes			0.051 (0.004 to 0.098)*		0.052 (0.007 to 0.098)*	
Feeding tube, No	Ref	Ref				
Yes	0.113 (0.014 to 0.21)*	0.104 (0.011 to 0.197)*				
Weight gain, No		Ref				
Yes		− 0.038 (− 0.076 to − 0.000)*				

*p < 0.05

**p < 0.001

## Discussion

The results of this paper provide detailed insight into which factors are associated with (changes in) SCNs in the recovery phase after HNC treatment, identifying groups of HNC patients that show limited improvement or worsening of SCNs over time and identifying groups of HNC patients with high absolute SCNs. SCNs in the domain of PDL, PSY, and HSIPS lowered significantly over time, whereas SEX needs remained constant. The highest unmet SCNs were reported on items of the domains on PDL, HSIPS, and HNC-specific functioning. Personal (especially coping style), clinical (especially tumor stage), psychological (especially anxiety and fear of cancer recurrence), lifestyle (especially smoking), and cancer-related quality of life factors (especially role functioning, social functioning, insomnia, trouble with social contact, painkiller usage, feeding tube usage, and weight gain) were associated with changes in SCNs over time and/or the absolute level of SCNs at 2 years follow-up.

When we compare the factors which were significantly associated with the course of SCNs over time in the current study (factors as assessed 6 months after treatment) to the results of our previous study on these factors as assessed at time of diagnosis, some similarities and differences are noteworthy [8]. A similar finding is that worse changes in SCNs over time (SEX, PDL, PSY, and PSY needs, respectively) were associated with the type of treatment (multimodality treatment), lower levels of oral pain, lower levels of fear of cancer recurrence, and lower levels of physical functioning as measured both at time of diagnosis and 6 months after treatment. This indicates that these factors should be considered when aiming to identify groups of HNC patients with a worse course of SCNs over time. A difference in findings is that insomnia, trouble with social contact, feeding tube usage, and weight gain at 6 months after treatment were associated with changes in 2 or more SCNs domains over time, whereas their baseline values were not associated with changes in SCNs over time. An explanation for this difference may be that these symptoms mostly arise during or after HNC treatment. Another difference is that tumor location was associated with changes in three SCNs domains from HNC diagnosis up to 2 years follow-up, whereas it was not associated with changes in SCNs from 6 months follow-up onwards. A previous prospective longitudinal study by Brennan et al. [16] also found that tumor location was not associated with changes in SCNs from 1 to 3 years after treatment. It is likely that at time of diagnosis, tumor location has predictive power for subsequent changes in SCNs, whereas, after cancer treatment, side effects of treatment are more important.

Besides changes in SCNs over time, the current study also investigated which factors at 6 months after treatment are associated with the level of SCNs at 2 years follow-up. In line with previous studies, we found that a higher level of psychological symptoms, a lower level of quality of life functioning, and a higher level of cancer-related quality of life symptoms are associated with higher SCNs [3, 4, 7, 8, 10, 18, 19]. Living status and multimodality treatment were, in contrast to previous studies [8, 15, 19], not associated with SCNs in our study. A potential explanation may be that we covered a broader spectrum of possible associated factors than earlier studies including detailed insight into cancer-related quality of life factors, social support, and personality, which might be more dominantly associated with SCNs. Our findings on the effect of personality (neuroticism, extraversion, and openness) are not reflected by earlier research and bring new insight. Neuroticism was associated with a higher level of psychological and health system information and patient support needs, extraversion with higher levels of health system information and patient support needs, and openness with lower levels of HNC-specific functioning needs. Corresponding findings were reported in patients with anxiety and depression disorders, where neuroticism was also associated with higher levels of healthcare needs [47].

This study is to our knowledge the largest longitudinal study focusing on supportive care needs from 6 months to 2 years after treatment. The unique NET-QUBIC cohort is a product of a collaboration of 9 (of the total 14) Dutch head and neck centers, providing data from patients throughout the Netherlands and reflecting different backgrounds. This provided us with a large cohort and gave us the opportunity to investigate a large amount of possible associated factors. This provided the power to investigate a wide range of demographic, personal, clinical, physical, psychological, social, lifestyle, and cancer-related quality of life factors in relation. A limitation of this study is that we did not collect HNC-specific SCNs at 6 months and 1 year after treatment and, therefore were unable to investigate changes in HNC-specific SCNs over time. Another limitation of this study is that we did not include data on actual supportive care use. In addition, we defined unmet needs from the patients’ perspective. This may have resulted in the identification of unmet needs for which no or no optimal supportive care options are available yet (e.g., unmet needs regarding dry mouth), and in some cases, patients may have already received care, but this may not have been perceived by the patient as sufficient. In other cases, patients may not have received care at all. Combined insight into SCNs and the actual use of supportive care is needed to provide clear recommendations for clinical practice. Also, no reference data is available on SCNs in the general population hampering the ability to draw conclusions on whether SCNs are a result of HNC and its treatment or a result of normal aging. Finally, no minimal clinically important differences are available for the SCNs domains, hampering the ability to determine if differences are besides statistically significant also of clinical importance.

Further research in the field of SCNs among HNC patients is warranted as we have shown that a large group of patients still present with moderate to high needs in the recovery phase of HNC treatment. It would be interesting to see if a further decrease in SCNs is seen in the years that follow. An interesting next step would be to develop a validated prediction model to further pinpoint which patients are in need at different time points after treatment. In the meantime, the findings of the current study may assist clinicians to improve their supportive care strategies targeting HNC patients. Although the SCNs decrease over time, up to 10% of the HNC patients still report an unmet need per item, and there is a large variety in the specific SCNs.

## Conclusion

SCNs in the recovery phase between 6 months and 2 years after treatment decrease over time. However, still 40.8% of all patients had 1 or more unmet needs. Several personal, clinical, psychological, lifestyle, and cancer-related quality of life factors were associated with SCNs. These results can be used to develop predictive models to personalize supportive care in the recovery phase after HNC treatment.

## Supplementary Information

Below is the link to the electronic supplementary material.

Supplementary file1 (DOCX 561 KB)

## Data Availability

The datasets generated and analyzed during the current study are not publicly available as the collection and integration of large amounts of personal, biological, genetic and diagnostic information precludes open access to the NET-QUBIC research data. Data are available from the corresponding author on reasonable request. On the NET-QUBIC website (www.kubusproject.nl) is described how NET-QUBIC data are made available for the research community.
