# Supplementary material for: Supportive care needs among head and neck cancer patients in the recovery phase from 6 months to 2 years after treatment: which factors matter?
Source: J Cancer Surviv. 2025 Feb 10;20(4):1538–49. doi: 10.1007/s11764-025-01753-0 (PMC13375755; doi:10.1007/s11764-025-01753-0)

**Appendix 1. Flow diagram**

^a^ Patients with no SCNs data between at either M12 or M24 were not coded as drop-out, as they could still participate in other parts of the study and could provide SCN data at a later time point.

| **Appendix 2. Changes in SCNs from 6 months follow-up to 2 years follow-up** | | |
| --- | --- | --- |
|  | Linear mixed model analysis | |
|  | Estimated mean change | 99% CI |
| 6-month follow-up  Physical and daily living  Psychological  Sexuality  Health system, information and patient support | ref. | ref. |
| 12-month follow-up  Physical and daily living  Psychological  Sexuality  Health system, information and patient support | -1.5  -1.9  -0.1  -3.7 | -3.7 to 0.7  -4.0 to 0.2  -2.6 to 2.4  -6.1 to -1.2 |
| 24-month follow-up  Physical and daily living  Psychological  Sexuality  Health system, information and patient support | -2.2  -3.6  -1.8  -6.3 | -4.5 to 0.2  -5.8 to -1.4  -4.5 to 0.8  -8.9 to -3.7 |
|  | | |

| **Appendix 3. Percentage of HNC patients with moderate to high unmet needs per item at M6, M12 and M24** | | | |
| --- | --- | --- | --- |
| **SCNS domains and items** | **% moderate-high unmet needs M6** | **% moderate-high unmet needs M12** | **% moderate-high unmet needs M24** |
| ***Physical & daily living needs*** |  |  |  |
| Pain | 7.3% | 6.3% | 4.7% |
| Lack of energy/tiredness | 11.8% | 9.7% | **10.8%** |
| Feeling unwell a lot of the time | 4.2% | 4.0% | 2.6% |
| Work around the home | 6.7% | 5.4% | 6.8% |
| Not being able to do the things you used to do | 6.5% | 6.0% | 6.6% |
| ***Psychological needs*** |  |  |  |
| Anxiety | 5.2% | 3.6% | 2.3% |
| Feeling down or depressed | 5.0% | 4.5% | 4.1% |
| Feelings of sadness | 5.2% | 5.8% | 3.8% |
| Fears about the cancer spreading | 10.2% | 8.8% | 5.8% |
| Worry that the results of treatment are beyond your control | 6.8% | 6.3% | 4.6% |
| Uncertainty about the future | 8.6% | 7.8% | 6.6% |
| Learning to feel in control of your situation | 8.8% | 6.3% | 4.9% |
| Keeping a positive look | 7.3% | 7.8% | 5.5% |
| Feelings about death and dying | 6.9% | 5.5% | 4.9% |
| Concerns about the worries of those close to you | 10.8% | 9.8% | 6.1% |
| ***Sexuality needs*** |  |  |  |
| Changes in sexual feelings | 6.6% | 6.9% | 5.6% |
| Changes in your sexual relationships | 6.2% | 6.9% | 6.1% |
| To be given information about sexual relationships | 5.3% | 5.1% | 4.4% |
| ***Health system information and patient support needs*** |  |  |  |
| More choice about which cancer specialist you see | 3.8% | 3.0% | 2.3% |
| Reassurance by medical staff that the way you feel is normal | 8.1% | 5.5% | 4.7% |
| Hospital staff attending promptly to your physical needs | 5.0% | 5.5% | 4.6% |
| Hospital staff acknowledging, and showing sensitivity to, your feelings and emotional needs | 5.0% | 4.8% | 4.1% |
| Being given written information about the important aspects of your care | 6.7% | 6.0% | 3.8% |
| Being given information (written, diagrams, drawings) about aspects of managing your illness and side-effects at home | 6.6% | 5.8% | 7.0% |
| Being given explanations of those tests for which you would like explanations | 9.1% | 6.5% | 0.0% |
| Being adequately informed about the benefits and side-effects of treatments before you choose to have them | 9.6% | 7.3% | 4.4% |
| Being informed about your test results as soon as feasible | **13.5%** | **11.6%** | 7.0% |
| Being informed about cancer which is under control or diminishing | **17.5%** | **11.8%** | **8.4%** |
| Being informed about things you can do to help yourself get well | **14.1%** | 10.1% | **8.4%** |
| Having access to professional counselling (e.g., psychologist, social worker, counsellor, nurse specialist) if you, family or friends need it | 9.4% | 5.8% | 6.1% |
| Being treated like a person not just another case | **12.4%** | **11.6%** | **8.5%** |
| Being treated in a hospital or clinic that is as physically pleasant as possible | 10.6% | **10.4%** | 7.0% |
| Having one member of hospital staff with whom you can talk to about all aspects of your condition, treatment and follow-up | **14.0%** | **13.4%** | **9.7%** |
| ***HNC-specific functioning needs*** |  |  |  |
| Problems with chewing and or swallowing | N.A. | N.A. | 4.8% |
| Problems with dry mouth and/or sticky mucus | N.A. | N.A. | **8.4%** |
| Problems with weight (underweight or overweight) | N.A. | N.A. | 3.0% |
| To be informed on nutrition | N.A. | N.A. | 3.0% |
| Difficulty speaking | N.A. | N.A. | 3.0% |
| Problems with hearing | N.A. | N.A. | 3.6% |
| Oral hygiene | N.A. | N.A. | 0.0% |
| Problems with mobility of neck or shoulders | N.A. | N.A. | 7.1% |
| ***Lifestyle needs*** |  |  |  |
| Quit smoking | N.A. | N.A. | 3.6% |
| Quit drinking | N.A. | N.A. | 1.2% |
| ***Single item*** |  |  |  |
| More choice about which hospital you attend | 1.5% | 1.8% | 2.0% |
| Care of your stoma and/or voice prosthesis | 0.0% | 2.0% | 0.0% |
| Top 5 moderate to high unmet needs per item in **bold.** | | | |

| **Appendix 4. Univariate association (*p-value*) between fixed and post-treatment (6 months follow-up) factors and changes in supportive care needs from 6 to 2 years follow-up** | | | | |
| --- | --- | --- | --- | --- |
|  | Physical and daily living | Psychological | Sexuality | Health system, information and patient support |
| **Demographic** |  |  |  |  |
| Age | 0.598 | 0.250 | 0.877 | 0.509 |
| Gender | 0.336 | 0.324 | 0.987 | 0.400 |
| Living arrangement | 0.405 | 0.567 | 0.378 | 0.706 |
| Educational level | 0.684 | 0.691 | 0.325 | 0.580 |
| Employment status (baseline) | 0.494 | 0.167 | 0.553 | 0.316 |
| Self-efficacy | 0.174 | 0.663 | 0.805 | 0.285 |
| **Personal** |  |  |  |  |
| Personality  Neuroticism  Extraversion  Openness  Agreeableness  Conscientiousness | 0.454  0.762  0.800  0.608  **0.046** | 0.820  0.190  0.474  0.282  0.172 | 0.091  0.464  0.677  0.080  0.462 | 0.853  0.241  0.771  0.568  0.820 |
| Coping style  Active tackling  Palliative reacting  Avoiding  Seeking social support  Passive reacting  Expression of emotions  Reassuring thoughts | **0.013**  0.776  0.483  0.816  0.823  0.755  0.590 | 0.064  **0.031**  0.373  0.246  0.499  0.672  0.215 | 0.585  0.701  0.226  0.204  0.893  0.211  0.919 | 0.656  0.172  0.831  **0.046**  0.087  0.703  0.366 |
| **Social** |  |  |  |  |
| Social support | 0.707 | 0.567 | 0.797 | 0.969 |
| **Lifestyle** |  |  |  |  |
| BMI | 0.537 | 0.165 | 0.938 | 0.624 |
| Muscle strength | 0.258 | 0.315 | 0.090 | 0.407 |
| Smoking | 0.440 | 0.744 | 0.265 | 0.956 |
| Alcohol | 0.172 | 0.764 | 0.700 | 0.214 |
| **Psychological** |  |  |  |  |
| Anxiety | 0.585 | **0.012** | 0.134 | 0.140 |
| Depression | 0.523 | 0.399 | 0.052 | **0.025** |
| Fear of recurrence | 0.076 | **<0.001** | 0.102 | **0.005** |
| **Clinical** |  |  |  |  |
| Type of treatment | 0.953 | **0.008** | 0.064 | 0.228 |
| Tumor stage | 0.558 | **0.010** | 0.381 | **0.031** |
| Tumor site | 0.903 | 0.207 | 0.123 | 0.935 |
| Comorbidities | 0.415 | 0.842 | 0.918 | 0.675 |
| WHO performance | 0.387 | 0.940 | 0.233 | 0.317 |
| **EORTC** |  |  |  |  |
| Global quality of life | 0.360 | 0.813 | 0.321 | 0.057 |
| Physical functioning | 0.060 | 0.156 | 0.590 | **0.013** |
| Role functioning | 0.352 | 0.268 | **0.038** | 0.050 |
| Emotional functioning | 0.242 | **0.009** | 0.284 | 0.164 |
| Cognitive functioning | 0.244 | 0.085 | **<0.001** | 0.170 |
| Social functioning | **0.005** | 0.290 | 0.066 | **0.012** |
| Fatigue | **0.009** | 0.433 | 0.273 | 0.090 |
| Nausea and vomiting | 0.555 | 0.327 | 0.459 | 0.245 |
| Pain | 0.251 | 0.411 | 0.724 | 0.114 |
| Dyspnea | 0.143 | 0.670 | 0.407 | 0.712 |
| Insomnia | 0.566 | 0.603 | 0.557 | 0.367 |
| Appetite loss | 0.759 | 0.142 | **0.043** | 0.687 |
| Constipation | 0.091 | **0.029** | **0.041** | 0.879 |
| Diarrhea | 0.107 | 0.475 | **0.025** | 0.743 |
| Financial problems | 0.067 | 0.231 | 0.223 | 0.734 |
| **HNC symptoms** |  |  |  |  |
| Oral pain | **0.002** | 0.185 | 0.620 | **0.012** |
| Swallowing | 0.102 | 0.316 | **0.049** | **0.047** |
| Senses problems | 0.749 | 0.323 | 0.987 | 0.353 |
| Speech problems | **0.014** | **0.034** | 0.381 | **0.005** |
| Trouble with social eating | **0.021** | 0.523 | 0.142 | **0.004** |
| Trouble with social contact | **0.002** | 0.086 | **0.040** | **0.002** |
| Less sexuality | 0.082 | 0.294 | 0.299 | 0.634 |
| Teeth | 0.531 | 0.891 | 0.810 | 0.381 |
| Opening mouth | 0.414 | 0.055 | 0.832 | **0.025** |
| Dry mouth | 0.246 | 0.209 | 0.650 | 0.098 |
| Sticky saliva | 0.600 | 0.728 | 0.949 | 0.092 |
| Coughing | 0.459 | 0.756 | 0.283 | 0.436 |
| Felt ill | **0.044** | **0.034** | 0.400 | **0.034** |
| Pain killers | 0.423 | 0.576 | 0.417 | 0.935 |
| Nutritional supplements | 0.242 | 0.228 | 0.435 | **0.028** |
| Feeding tube | **0.024** | **0.015** | 0.750 | 0.213 |
| Weight loss | 0.086 | 0.680 | 0.907 | 0.674 |
| Weight gain | 0.053 | **0.022** | 0.115 | 0.430 |
| Significant associations are written in **bold.** | | | | |

**Appendix 5. The course of supportive care needs per baseline factor that was significantly found to be associated (factor*time)**

Supplementary material 2. The course of supportive care needs per baseline factor that was significantly found to be associated (factor*time)

Line graphs represent the average 0-100 score, in which a higher score indicate higher needs. For categorical factors the course is displayed per category. For continuous variable, the course is displayed for the mean score and +/- 1SD (or 0/100 if 1SD exceeded 0/100)

**Physical and daily living needs**


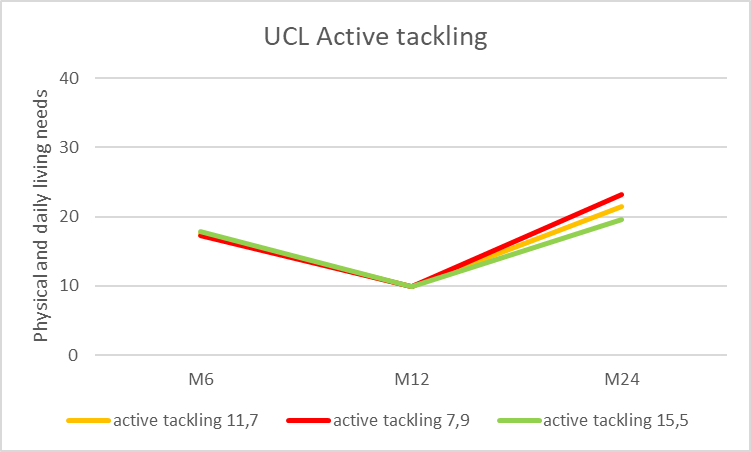

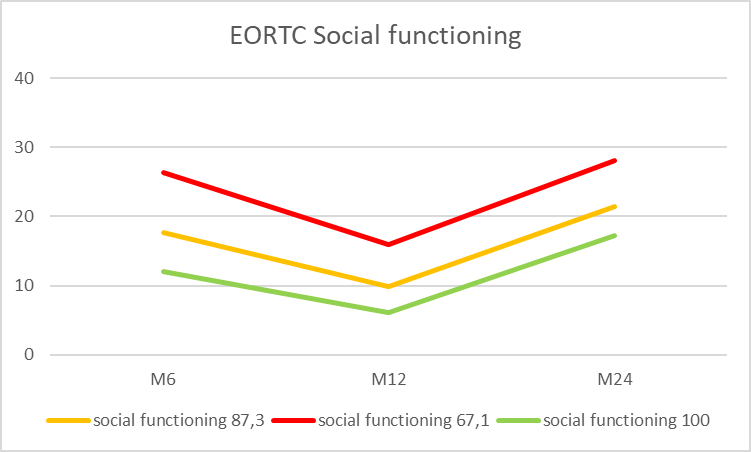


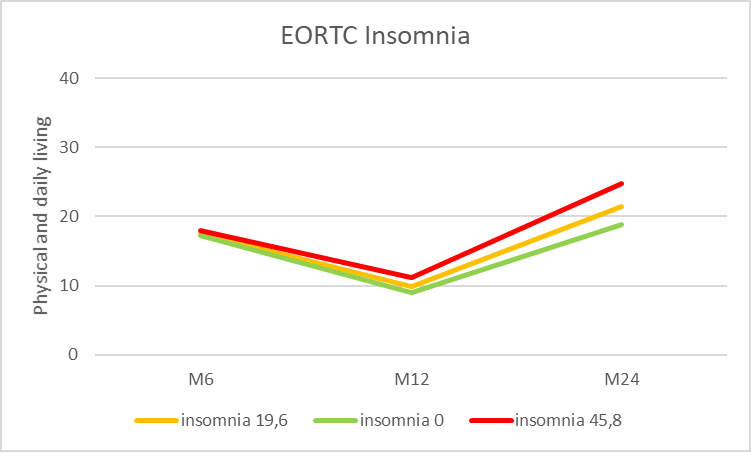

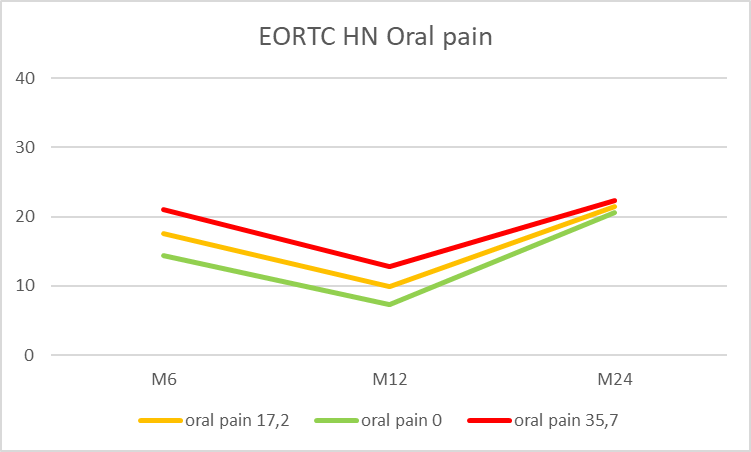


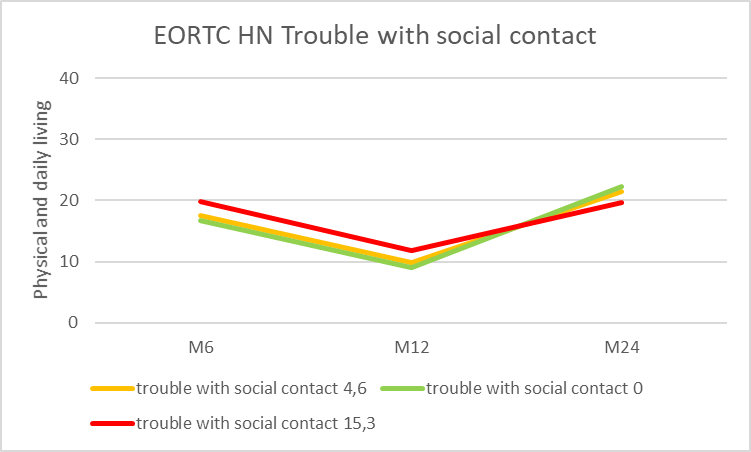

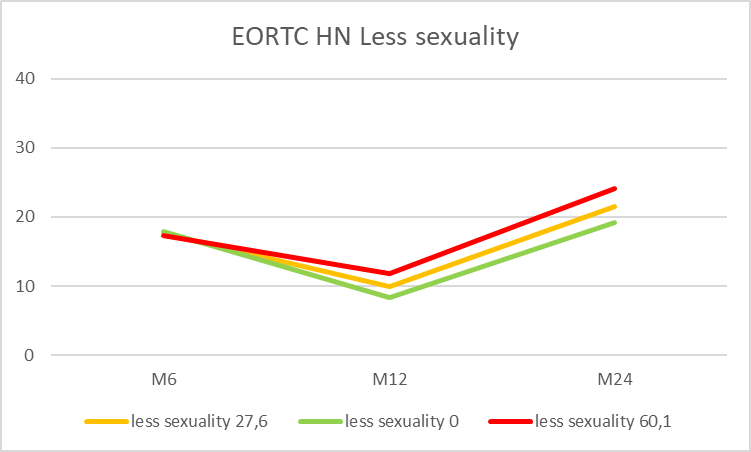


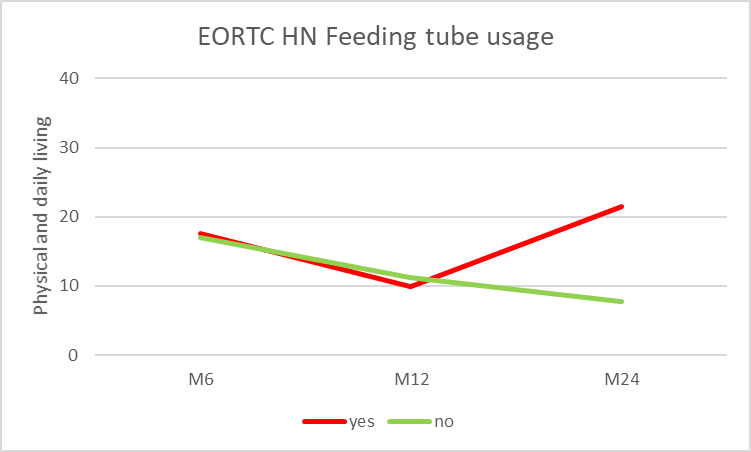

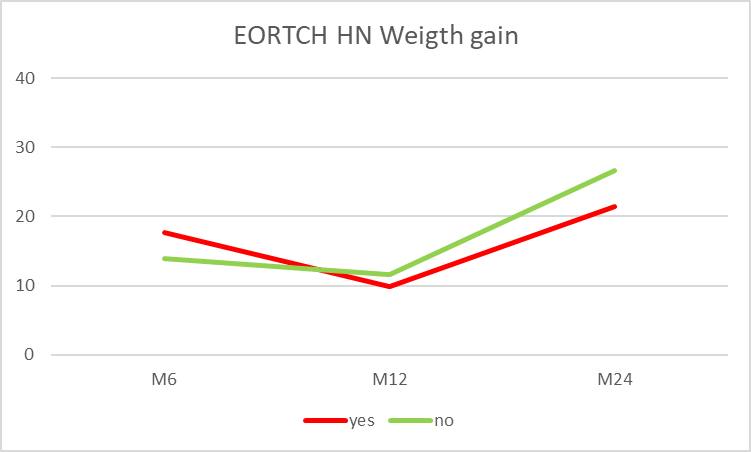


**Psychological needs**


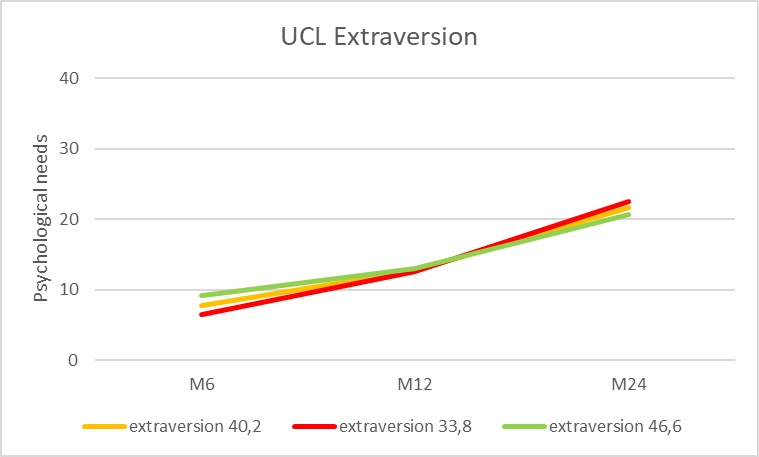

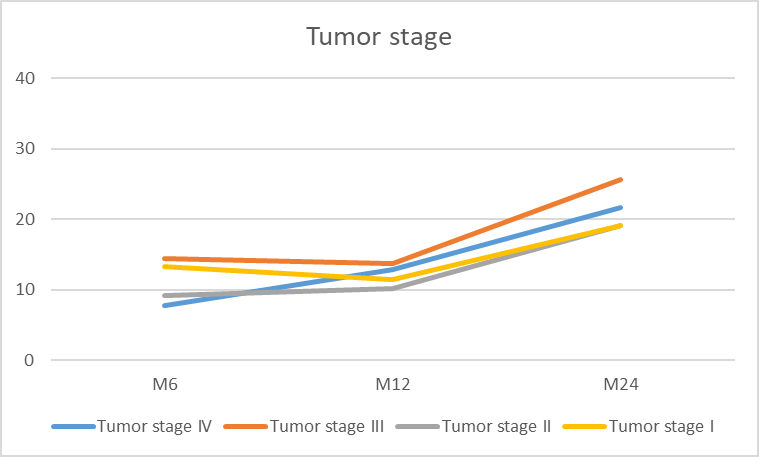


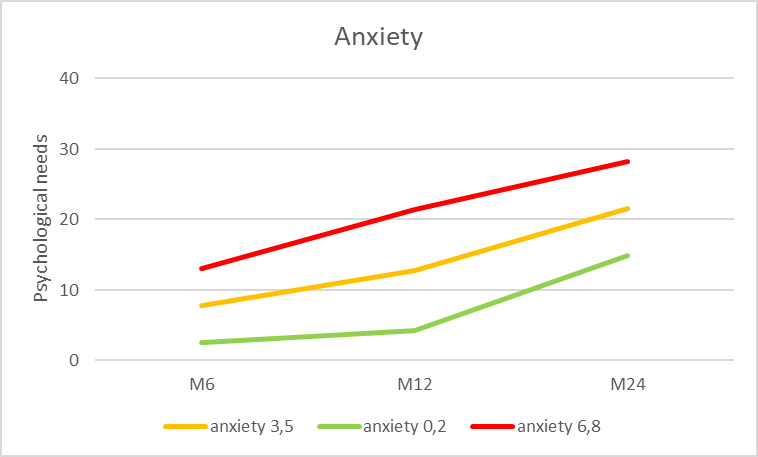

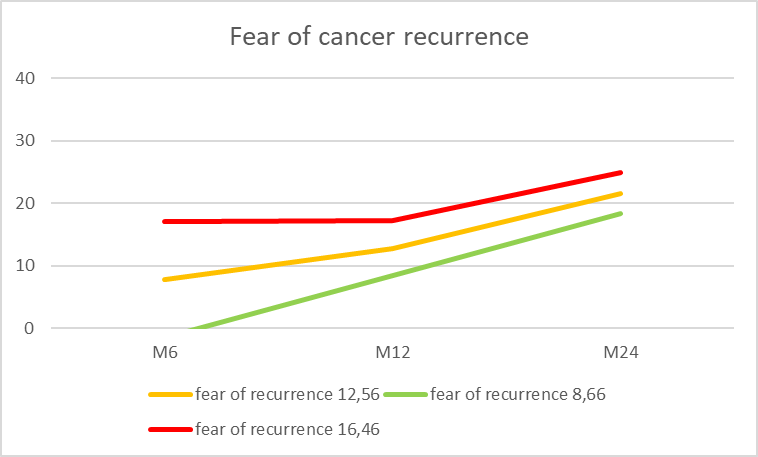


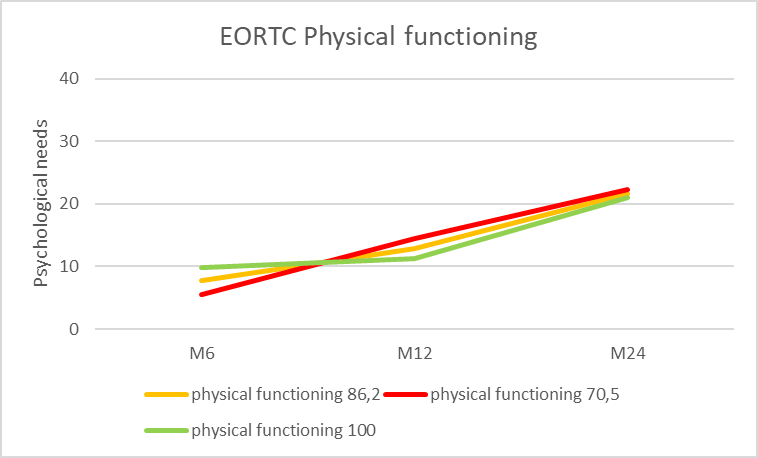

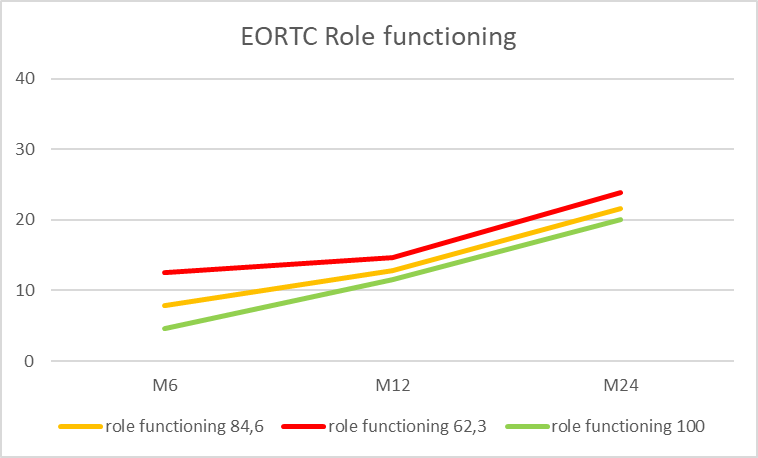


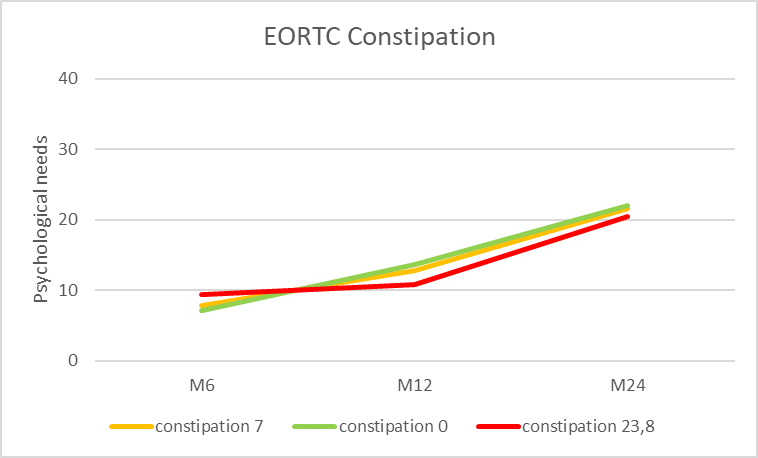

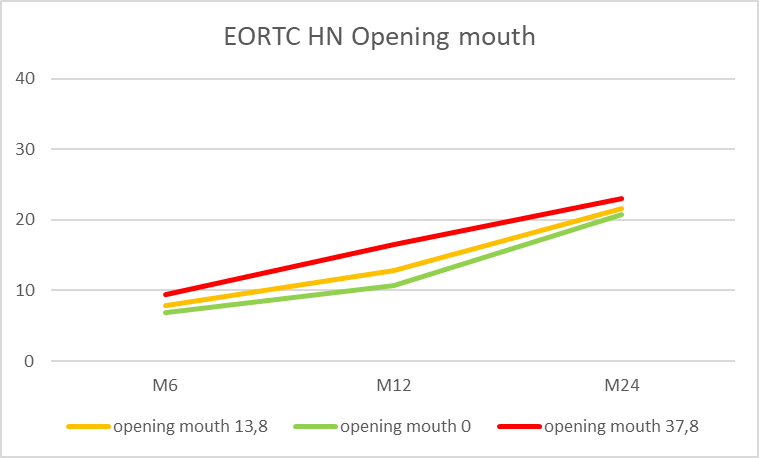


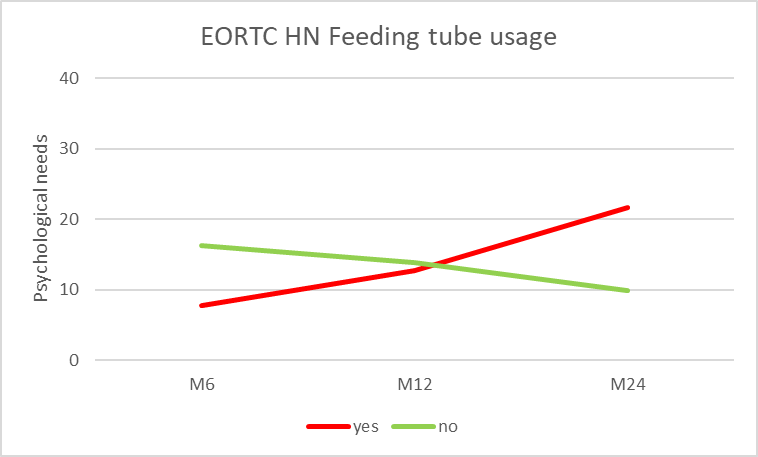

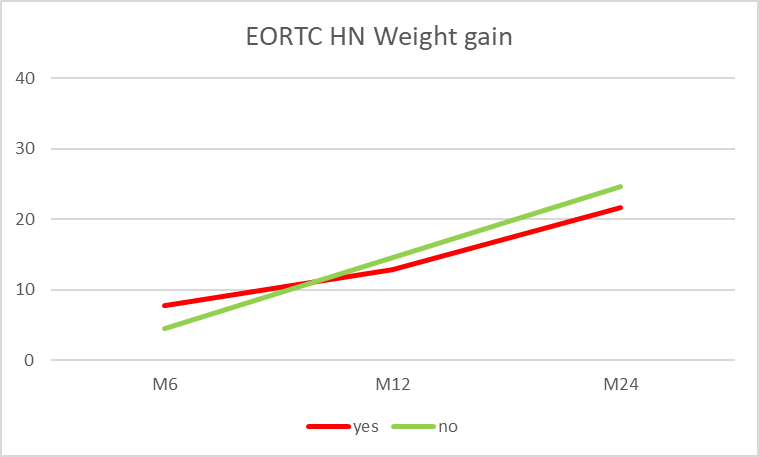


**Sexuality needs**


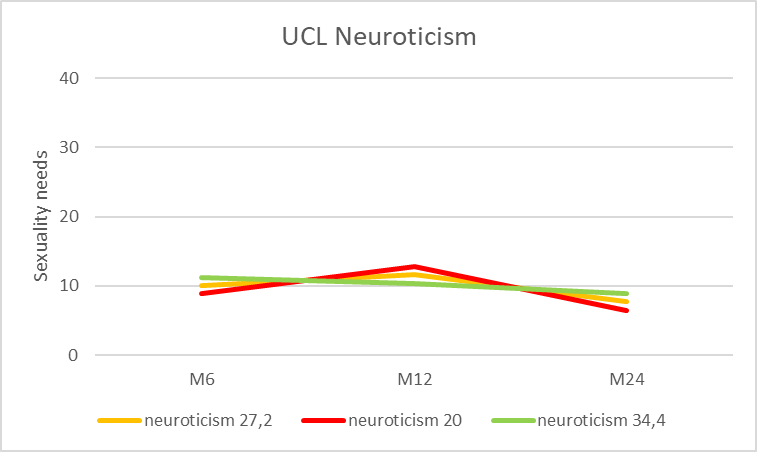

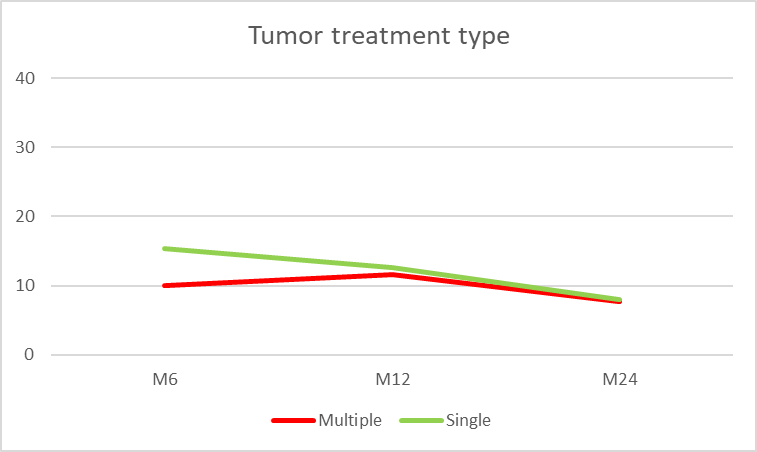


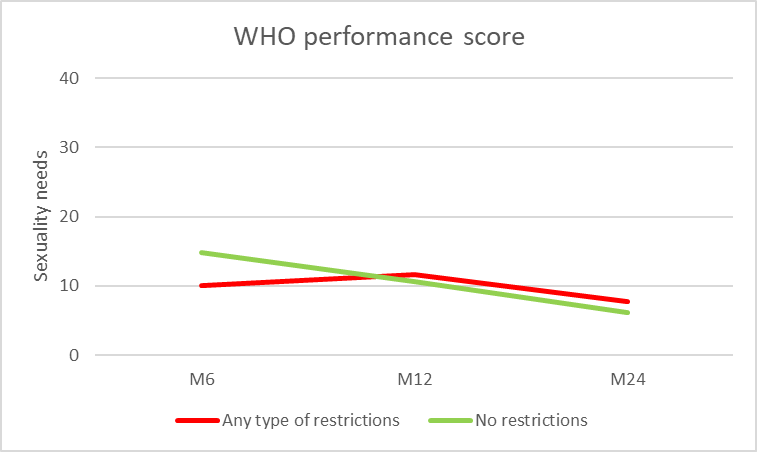

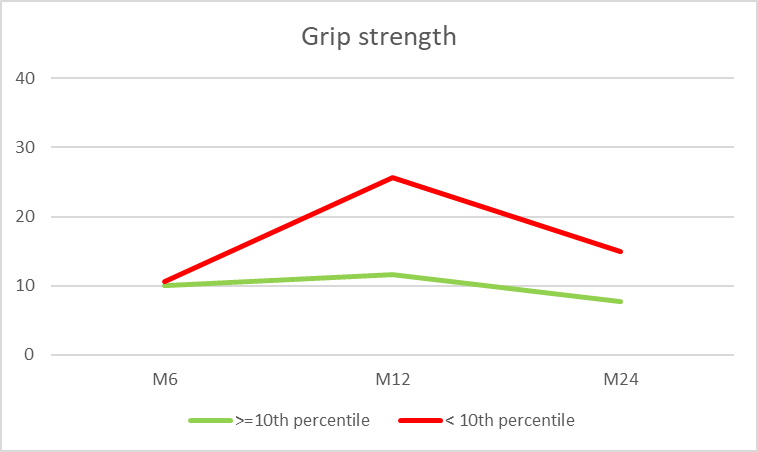


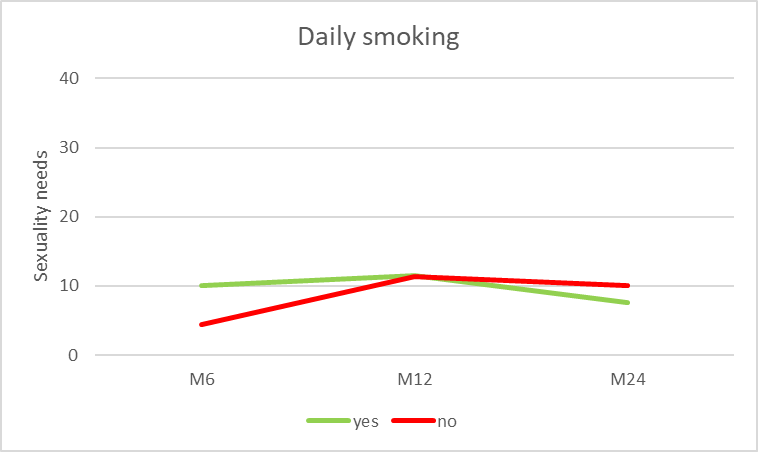

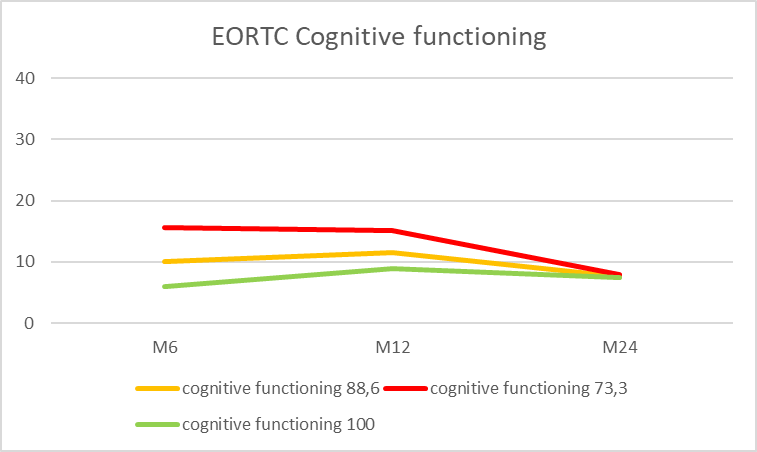


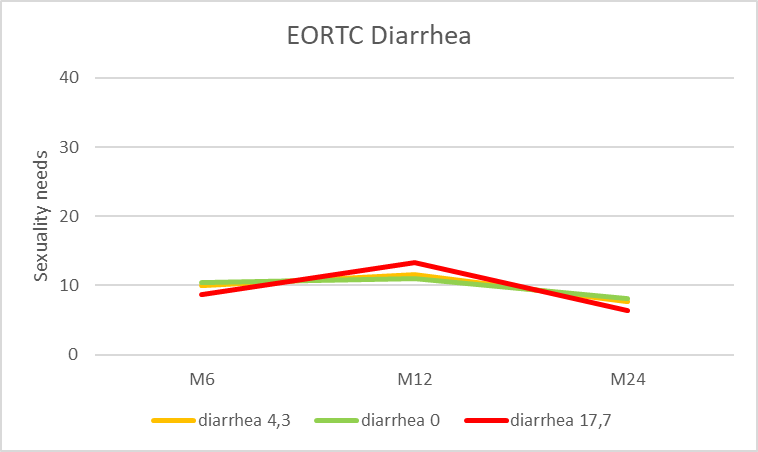


**Health system information and patient support needs**


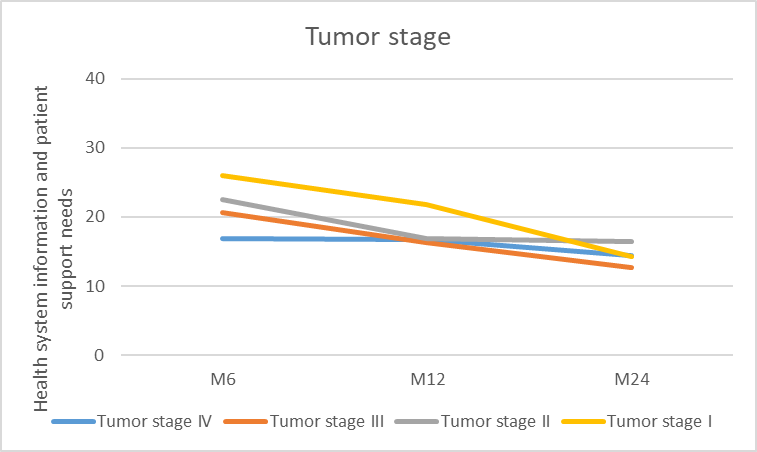

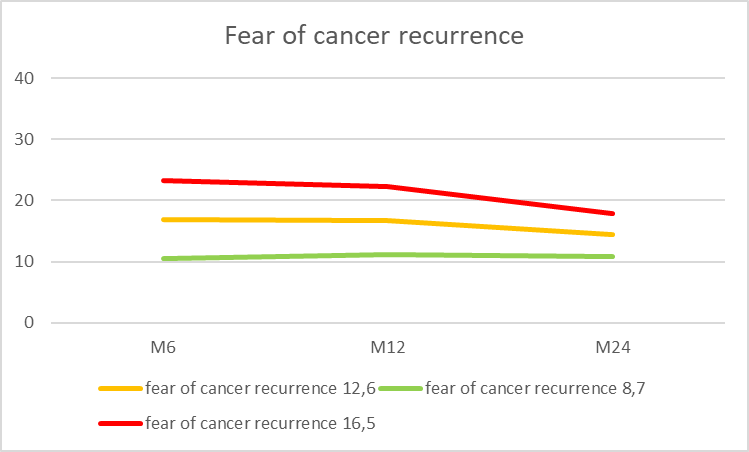


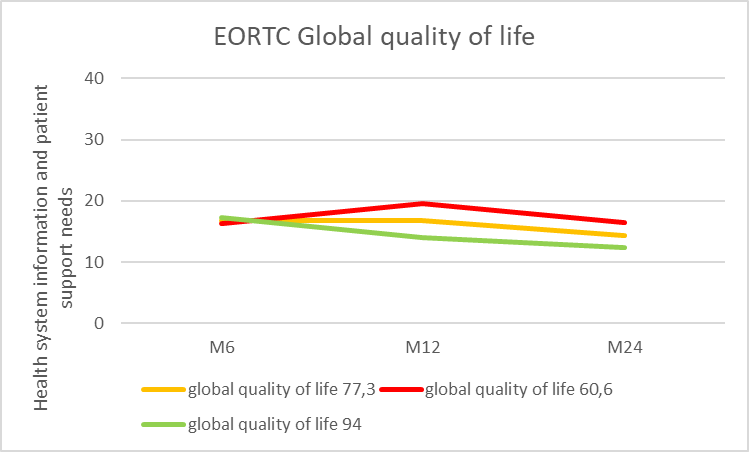

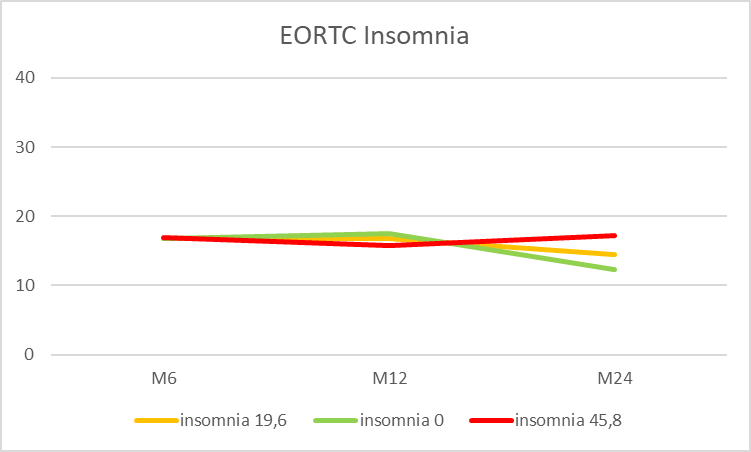


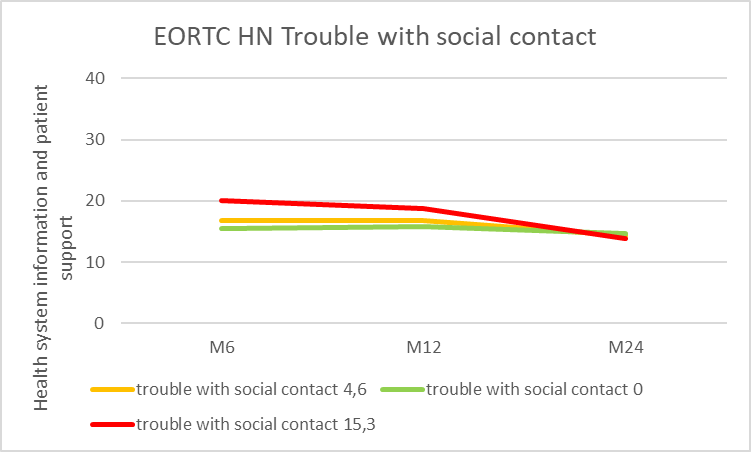

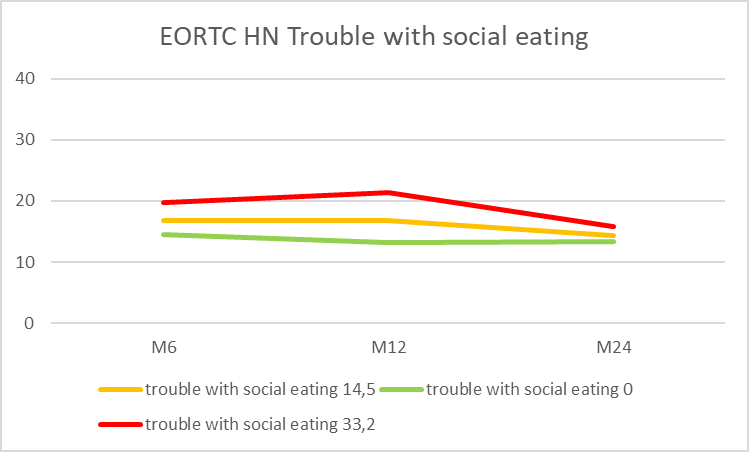


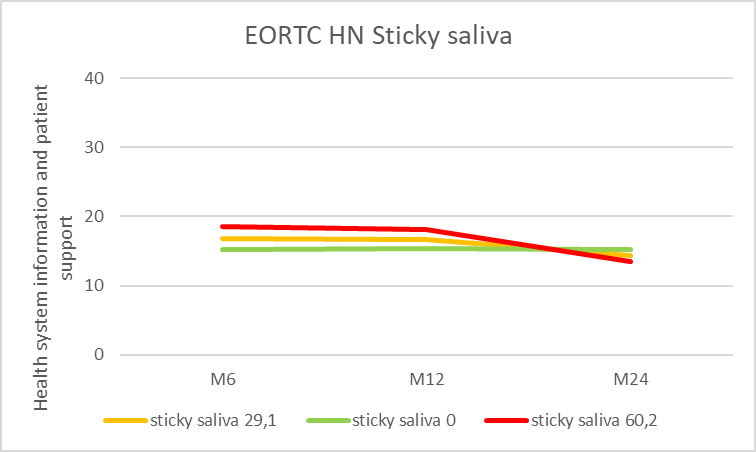

Supplement: Supplementary file 1 — Supplementary file1 (DOCX 561 KB) [file 11764_2025_1753_MOESM1_ESM.docx]
